# Detailed evaluation of sleep apnea using heart rate variability: a machine learning and statistical method using ECG data

**DOI:** 10.3389/fneur.2025.1636983

**Published:** 2025-09-10

**Authors:** Eyad Talal Attar

**Affiliations:** Department of Electrical and Computer Engineering, Faculty of Engineering, King Abdulaziz University, Jeddah, Saudi Arabia

**Keywords:** sleep apnea, heart rate variability, machine learning, nonlinear dynamics, autonomic nervous system, ECG, wearable diagnostics

## Abstract

**Background:**

Sleep apnea is a common sleep disorder associated with high degree of autonomic dysfunction and increased cardiovascular risk. Traditional diagnostic methods such as polysomnography (PSG) are costly, time-consuming, and sometimes unavailable. Heart rate variability (HRV), a noninvasively assessable measure, is another promising method for the assessment of autonomic perturbations during apneas. The objective of this study was to investigate the extent to which features derived from single-lead ECG are capable of differentiating apnea from non-apnea states in time-domain, frequency-domain and nonlinear HRV features.

**Methods:**

Analysis was done on 18 subjects from the PhysioNet Apnea-ECG database. After preprocessing to extract R-R intervals, the ECG signals were divided into 1-min epochs and classified as either apnea or non-apnea. Kubios software was used to extract HRV features, and one-way ANOVA was used for statistical comparison.

**Results:**

The predictability of HRV features was analyzed using machine learning classifiers Random Forest and XGBoost. Sympathetic markers (VLF and LF/HF) increased, while parasympathetic-related features (HF, RMSSD, SampEn) decreased during apnea (*p* < 0.05). Nonlinear features, including SampEn, showed high discriminatory performance (Cohen’s d = 2.93). The AUC of XGBoost model reached to 0.98, demonstrating the usefulness of the HRV features in precise apnea detection.

**Conclusion:**

HRV parameters can efficiently reflect autonomic disruption induced by SAAs, especially nonlinear and frequency domain indices. Augmented by machine learning, HRV analysis is a powerful and scalable technique toward real-time, non-invasive screening of sleep disordered breathing that can be implemented in to wearable health technology and digital sleep medicine.

## 1 Introduction

Sleep apnea is a frequent and potentially deadly sleep disorder that is not sufficiently recognized or treated across the world. It is defined by recurrent partial or complete obstruction of the upper airway during sleep, with drops in oxygen, breathing disturbances, and brief awakenings from sleep. Such events may impose severe stress on a number of physiological systems due to acute changes in intrathoracic pressure (ITP), broken sleep structure, and cyclical episodes of hypoxemia-re-oxygenation (1, 2). ARDS comes in two forms: central sleep Apnea (CSA) is caused by damage to the brain or spinal cord, which prevents the respiratory center from operating, and obstructive sleep apnea (OSA) is the closure of the airway at the back of the throat during sleep. Most are attributed to OSA, which is relatively common in middle-aged and older adults (3, 4). Obesity, alcohol consumption, and craniofacial anatomy are the commonly associated modifiable risk factors for OSA. There are important clinical consequences of sleep apnea. A chain of pathophysiological mechanisms, such as systemic inflammation, oxidative stress, endothelial dysfunction, and autonomic nervous system (ANS) imbalance, is initiated as a result of cyclic episodes of nocturnal hypoxia and sleep fragmentation (5–7). These changes are also associated with several of cardiometabolic symptoms (systemic hypertension, coronary artery disease, heart failure, insulin resistance, metabolic syndrome, and cerebrovascular accident (CVA)) (8–10). Additionally, sleep apnea is often related to mood disorders, such as depression, cognitive dysfunction, all-cause mortality, and sudden cardiac death (11, 12). Recent studies have explored the use of deep learning for HRV-based sleep apnea severity estimation, highlighting the potential of advanced models in capturing subtle physiological patterns for disease stratification. Integrating such approaches with our HRV framework may further enhance predictive performance and clinical relevance (13). Since the disease is asymptomatic during waking hours and the standard diagnostic method, namely, overnight polysomnography (PSG), is a time-consuming examination, however, diagnosis remains arduous even with these serious health-related issues. Although PSG is the reference standard, it is expensive, time-consuming, and not universally accessible, particularly in low-resource areas. For these reasons, an urgent need to find less expensive and more accessible diagnostic alternatives than the current PSG that accurately reflect the pathological changes associated with sleep apnea was prompted. One promising such surrogate is Heart Rate Variability (HRV), which is widely used as a non-invasive index of ANS activity. HRV refers to the cyclic variations in the time intervals between consecutive heartbeats (14–16). HRV provides important information on the autonomic disorder that sleep apnea syndrome is. One such potential surrogate is heart rate variability (HRV), the variability of the intervals between consecutive heartbeats, which has been utilized as a non-invasive measure of the ANS activity (14–16). HRV offers useful information about the autonomic deregulation inherent to sleep apnea. In the apnea phase, hypoxia and arousal induce sympathetic activation and vagal withdrawal, reflected in modified HRV profiles. A large number of analytical domains are available for assessing these autonomic shifts: frequency-domain parameters (e.g., VLF, LF, HF, and LF/HF) characterize the way autonomic power is distributed over different frequency bands, time-domain profile (e.g., SDNN, RMSSD, pNN50) represents the complexity of overall variability, and nonlinear parameters (e.g., ApEn, SampEn, SD1, SD2) characterize signal complexity and irregularity that offer a deeper understanding of the dynamic behavior of the cardiovascular system (17–22). The HRV features and definition used in this study are displayed in Table 1.

**Table 1:** Summary of heart rate variability (HRV) features across time domain, frequency domain, and nonlinear methods.

Feature	Unit	Description
Time domain features		
SDNN	ms	Standard deviation of all N.N. (normal-to-normal) intervals
rMSSD	ms	Root mean square of successive differences between adjacent N.N. intervals
pNN50	%	Percentage of successive N.N. intervals differing by more than 50 ms
HRV Triangular Index	-	Ratio of the total number of all N.N. intervals to the height of the histogram
Frequency domain features		
VLF	ms^2^	Very Low-Frequency power of HRV (0.0033–0.04 Hz)
LF	ms^2^	Low-Frequency power of HRV (0.04–0.15 Hz)
HF	ms^2^	High-Frequency power of HRV (0.15–0.4 Hz)
LF/HF Ratio	-	Ratio of low-frequency power to high-frequency power
Nonlinear features		
SD1	ms	Poincaré plot standard deviation perpendicular to the line of identity
SD2	ms	Poincaré plot standard deviation along the line of identity
ApEn	-	Approximate entropy, quantifies regularity and complexity
SampEn	-	Sample entropy, measures signal complexity

HRV changes in sleep apnea have been the subject of numerous studies, and the results consistently show elevated sympathetic indices (like LF and LF/HF ratio) and decreased parasympathetic markers (like HF and RMSSD). However, the existing literature is heterogeneous, with varying findings attributed to small sample sizes, inconsistent methodologies, and insufficient control for confounding variables (23–25).

Additionally, while frequency-domain analyses are more commonly employed, nonlinear HRV techniques—which are potentially more sensitive to subtle autonomic disturbances—remain underutilized. Furthermore, few studies have employed comprehensive statistical analyses to determine the reliability and discriminative power of HRV features across apnea and non-apnea conditions (26, 27).

In response to these gaps, the present study undertakes a comprehensive and statistically rigorous analysis of HRV metrics derived from ECG recordings of individuals experiencing sleep apnea. This investigation spans time-domain, frequency-domain, and nonlinear domains to capture a multidimensional view of autonomic modulation. Standardized techniques, including Fast Fourier Transform (FFT) and entropy-based algorithms, are utilized to extract HRV features, while robust statistical tests—such as one-way ANOVA—are applied to evaluate the significance of changes between control and apnea states.

The ultimate objective of this study is to identify HRV features that are consistently associated with autonomic disruption during apnea episodes to improve the use of HRV as a diagnostic and monitoring tool in sleep medicine. The combination of classical and novel HRV indices offers new perspectives to study the cardiovascular-autonomic interactions during SDB. Furthermore, the results corroborate the increasing interest of HRV-based metrics for digital health applications, e.g., wearable biosensors and mobile health platforms, which provide scalable, non-invasive and real-time measurement of the SA severity in an out of clinic scenario.

## 2 Methods

### 2.1 Data source and subject selection

This study utilized the publicly available Apnea-ECG database from PhysioNet, which includes annotated single-lead ECG recordings from adults undergoing overnight monitoring for suspected sleep apnea (26, 27). To determine whether apnea or hypopnea episodes were present or absent, clinical professionals labeled each ECG signal minute by minute after it was sampled at 100 Hz with 16-bit resolution.

This study chose a subset of 18 participants from the initial 35 based on the following standards to guarantee data dependability and clinical relevance: An apnea-hypopnea index (AHI) of more than five events per hour is considered clinically significant apnea, as is a minimum signal duration of 8 h to capture adequate variability across sleep cycles and adequate signal quality for precise R-peak detection.

With a mean age of 45 and a mean BMI of 28 kg/m^2^, the final cohort was deemed overweight, a known risk factor for obstructive sleep apnea (OSA) (20). This selection ensured the inclusion of individuals likely to exhibit meaningful autonomic dysfunction during apnea episodes.

### 2.2 Signal preprocessing

Strong signal preprocessing was required in order to minimize noise and to optimize HRV analysis precision. A notch filter was initially applied to suppress powerline interference at 50 Hz, then subsequently high-pass filtered for removal of the baseline wander. Moreover, the R-peak detection was performed based on the threshold algorithm built on the Welch periodogram that approximates the power spectral density, in order to improve detection precision, particularly in long-duration, noisy and morphologically varied recordings (10). Following R-peak detection, R-R intervals were computed to construct the HRV time series. The signals were segmented into 1-min, non-overlapping epochs—a resolution shown to effectively capture autonomic changes while maintaining computational efficiency (17). Each epoch was labeled as “apnea” or “non-apnea” based on corresponding clinical annotations. Epochs containing either apnea or hypopnea events were collectively labeled as apnea-positive, in line with the apnea-hypopnea index (AHI) used clinically.

### 2.3 HRV feature extraction

To comprehensively capture autonomic nervous system (ANS) activity, the study extracted HRV features across three primary domains using Kubios HRV Premium v2.2, a validated platform widely used in both research and clinical contexts (17).

These included SDNN (standard deviation of NN intervals), RMSSD (root mean square of successive differences), pNN50 (percentage of successive intervals differing by >50 ms), and the HRV Triangular Index. These features primarily reflect overall heart rate variability and vagal (parasympathetic) modulation. Reduced time-domain metrics during apnea indicate parasympathetic withdrawal—a pattern confirmed in this study.

Power within VLF (0–0.04 Hz), LF (0.04–0.15 Hz) and HF (0.15–0.4 Hz) was estimated using spectral decomposition analysis with Fast Fourier Transform (FFT). The LF/HF, as a measure of sympathovagal balance, was also determined (28, 29). Apneic events were generally marked by high sympathetic tone (as evidenced by increased LF/HF) and low parasympathetic modulation (represented by low HF), which was in agreement with our result.

The study included SD1 and SD2 (Poincaré plot descriptors) along with Approximate Entropy (ApEn) and Sample Entropy (SampEn). These metrics capture the complexity and irregularity of the R-R interval series. Entropy-based measures are especially sensitive to nonlinear and dynamic alterations in heart rate regulation (18, 30), which become dampened under stress or pathophysiological states like apnea. In our results, entropy features demonstrated high discriminative power and statistical significance, affirming their value.

### 2.4 Statistical analysis

One-way ANOVA analysis was used to determine whether or not the HRV features for the apnea epochs and the non-apnea epochs are different. The zero exposure hypothesis was that the mean feature value was the same across conditions. A *p*-value of 0.05 was used as the threshold of statistical significance. This validates the anticipated autonomic shifts during the apnea episodes, evidenced by large decreases in parasympathetically-associated variables (HF, SampEn) and increases in sympathetically- or stress-associated variables (VLF, LF/HF) (5). All analyses were implemented in MATLAB R2017a; enabling reproducibility and compatibility with preprocessing routines.

### 2.5 Machine learning classification

To explore the predictive utility of HRV features, we implemented a supervised machine learning framework. The labeled HRV dataset was divided into training (80%) and testing (20%) subsets using stratified random sampling to preserve class proportions.

Before training, feature standardization was applied (zero mean, unit variance). Four popular classifiers were evaluated due to their proven effectiveness in physiological data modeling Logistic Regression, Support Vector Machine (SVM), Random Forest, and XGBoost (34, 35).

Five-fold cross-validation performance on the training set was used to select the model. The performance was also calculated for test set’s metrics such as F1-score, AUC-ROC, recall, accuracy, and precision. In addition, nonlinear and spectral features (e.g., SampEn, VLF, HF) were important to classification accuracy as measured by feature importance scores in ensemble models, and aligned well with statistical findings as well as the physiology.

### 2.6 Integration of statistical and predictive insights

By combining traditional statistical methods with machine learning, this study bridges the gap between group-level inference and individual-level prediction. While ANOVA identified features with significant mean differences between apnea and control states, machine learning assessed their ability to discriminate apnea epochs in real time. This two-pronged strategy makes HRV analysis more comprehensible as well as more applicable.

Furthermore, the statistically significant statistically significant (and of course biologically meaningful) features (such as the decreased SampEn, increased VLF and LF/HF ratios) seem to have a high predictive power also, indicating not only significance but also practical usefulness of HRV metrics in screening and monitoring applications. These results provide the basis for the design of wearable, real-time diagnostic systems for sleep apnea, based on physiological and data-driven validation.

### 2.7 Ethical considerations

This study used data from the publicly available Apnea-ECG database hosted on PhysioNet (26, 27). The original data collection was approved by the Institutional Review Board (IRB) of the University of Quebec at Montreal (approval no. IRP-2001-10-02), with protocols also reviewed and approved at participating institutions including McGill University (Montreal, Canada) and CHU de Bordeaux—Hôpital du Haut-Lévêque (Pessac, France). All participants provided written informed consent prior to enrollment.

Prior to publication at PhysioNet, all data sets were anonymized and scrambled to ensure privacy protection of the patients. The study was conducted in accordance with the ethical principles of the Declaration of Helsinki for research involving human subjects. The original study, although not prospectively registered, was done in accordance with contemporary ethical standards when the trial was initiated. For more information on the Apnea-ECG database, visit PhysioNet at: https://physionet.org/content/apnea-ecg/1.0.0/.

## 3 Results

Table 2 summarizes the demographic characteristics of study participants. Data are presented as mean ± standard deviation, except for gender distribution.

**Table 2:** Subject demographics.

Parameter	Value
Gender	Male: 22 (63%) Female: 13 (37%)
Age	45 ± 1.8 years
Weight	86 ± 3.5 kg
Height	175 ± 0.9 cm
BMI	28 ± 1.0 kg/m^2^

### 3.1 Sleep apnea duration and indexes

The mean total duration of monitoring for the subjects is presented in Table 3 (491 ± 5.3 min). The average A.I. was 21.8 ± 4.0, and the average H.I. was 6.1 ± 1.6. Of that duration, 186 ± 29 min (approx. 38%) showed episodes of apnea.

**Table 3:** Subjects’ apnea information.

Apnea information	Data
Length	491 ± 5.3 min
Non-Apnea	305 ± 26 min
Apnea	186 ± 29 min
Hours with Apnea	5.0 ± 0.6 h
Apnea Index (A.I.)	21.8 ± 4.0
Hypopnea Index (H.I.)	6.1 ± 1.6
Apnea-Hypopnea Index (A.H.I.)	28 ± 4.6

### 3.2 HRV feature analysis

#### 3.2.1 Linear and non-linear HRV features

Summary mean HRV parameters during apnea and control are presented in Table 4. A prominent reduction in HF (11 1.0 vs. 8.4 1.0, *p* 0.05) and LF (24 2.0 vs. 18 1.7, *p* 0.05) was noted, denoting autonomic imbalance and parasympathetic withdrawal during apnea. Similarly, VLF (62 ± 2.4 vs.72 ± 2.4, *p* < 0.05) also increased significantly, representing a sympathetic activation. Finally, non-linear measures which demonstrate reduced data complexity with apnea are ApEn and SampEn (0.93 ± 0.04 and 0.8 ± 0.05, respectively).

**Table 4:** HRV attributes in control and apnea situations.

Feature	Control	Apnea	Trend
SDNN	98 ± 8.7	102 ± 13	↑
RMSSD	58 ± 4.8	53 ± 9.2	↓
pNN50	12 ± 3.1	11 ± 3.3	↓
RR Trin	25 ± 2.4	22 ± 2.6	↓
VLF	62 ± 2.4	72 ± 2.4^*^	↑
LF	24 ± 2.0	18 ± 1.7^*^	↓
HF	11 ± 1.0	8.4 ± 1.0^*^	↓
LF/HF	2.3 ± 0.2	2.5 ± 0.2	↑
SD1	41 ± 3.4	37 ± 6.4	↓
SD2	132 ± 12	140 ± 17	↑
ApEn	0.98 ± 0.03	0.95 ± 0.04	↓
SampEn	0.93 ± 0.04	0.8 ± 0.05	↓

^*^Significant difference between control vs. apnea (*p* < 0.05).

#### 3.2.2 Visualizations and statistical insights

**Figure 1:**
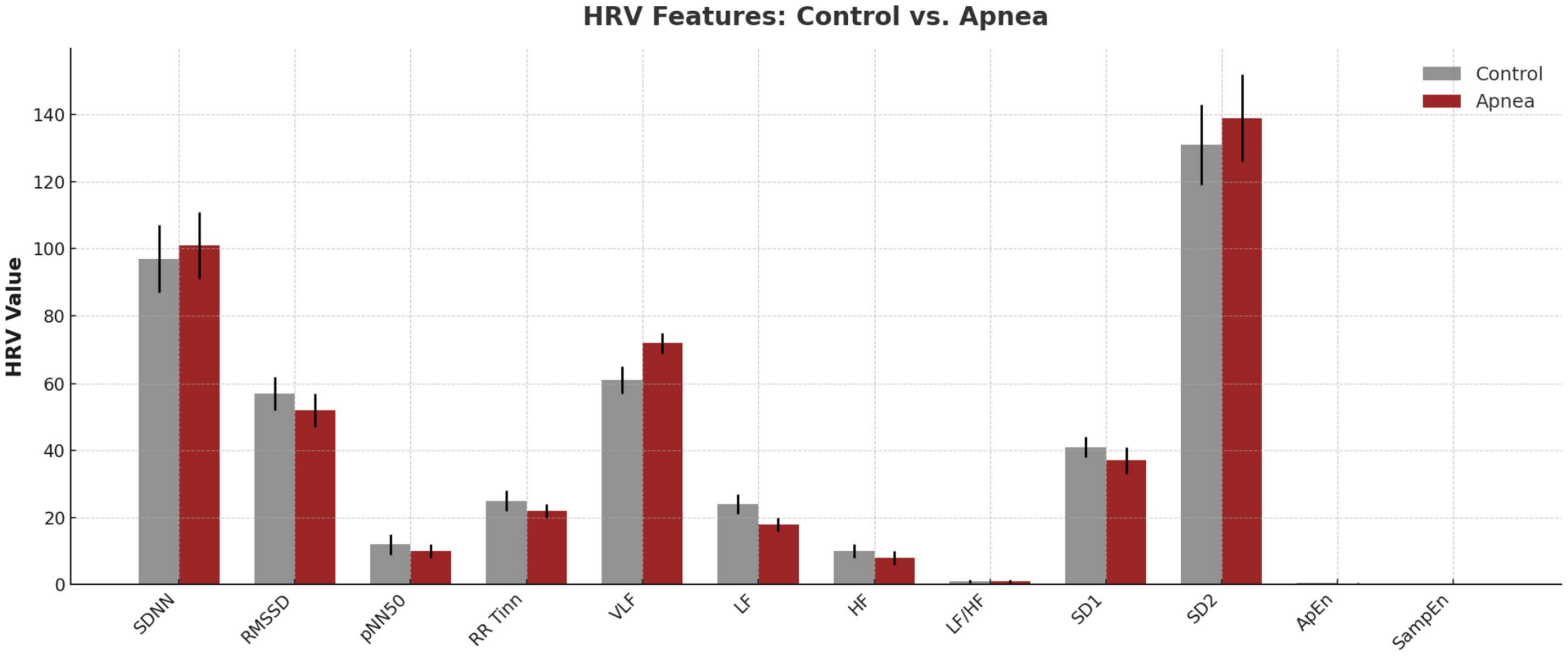
HRV features (control vs. apnea).

Figure 1 expresses relevant features of the HRVs plotted in a groupwise manner; statistically significant differences between controls and in apnea are shown. During apnea, LF and HF power decreased, and VLF power doubled—in line with previously described mechanisms regulating HF power and LF power in response to IHO.

**Figure 2:**
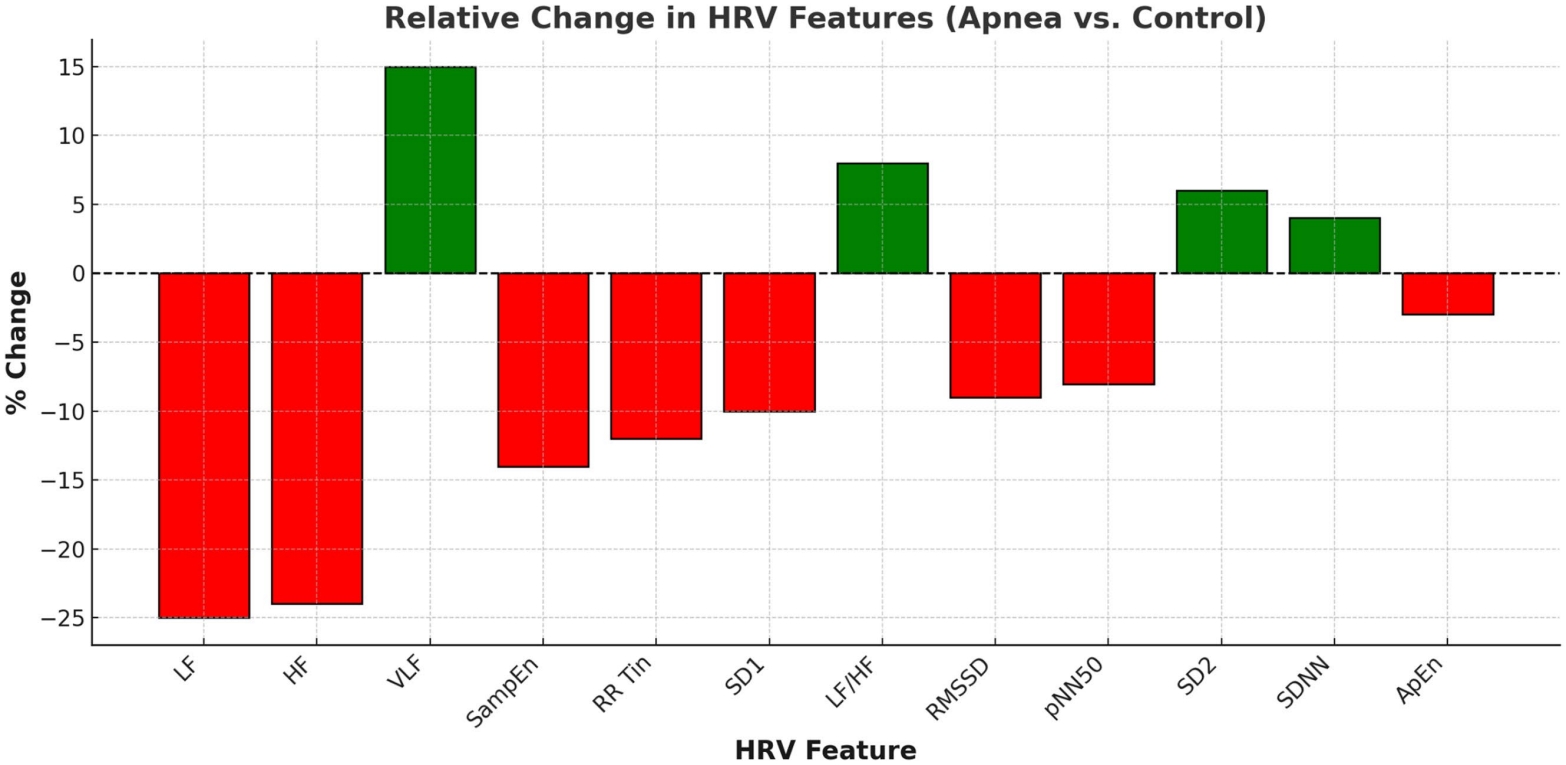
Relative change in HRV features (apnea vs. control).

Figure 2 illustrates the relative change in each HRV metric from control to apnea conditions. Metrics such as VLF and LF/HF ratio increased (green bars), while HF and RMSSD decreased (red bars), further confirming the autonomic shift.

Table 5 compares the calculated HRV measures during control and apnea. The pNN50, RR Triangular Index, VLF, LF, HF, LF/HF ratio, ApEn and SampEn differed significantly (*p* < 0.05) between the two groups. More importantly, VLF, LF, HF and SampEn reached large to very large effect sizes (Cohen’s d > 0.8), which provides evidence of a marked autonomic modulation during apnea episodes. The increase in VLF and decrease in HF and SampEn are consistent with heightened sympathetic activation and reduced parasympathetic and complexity-related modulation. These findings underscore the discriminative power of specific linear and nonlinear HRV parameters in differentiating apnea from non-apnea states.

**Table 5:** Comparison of heart rate variability (HRV) features between control and apnea groups.

Feature	Control mean ± SD	Apnea mean ± SD	*p*-value	Cohen’s d
SDNN	97.81 ± 6.50	101.01 ± 13.66	0.3505	−0.30
RMSSD	56.29 ± 4.55	54.38 ± 8.54	0.3825	0.28
pNN50	10.08 ± 3.20	12.35 ± 2.82	0.0225	−0.75
RR Trin	25.14 ± 1.99	21.82 ± 2.83	0.0001	1.36
VLF	61.79 ± 2.05	72.25 ± 2.59	<0.0001	−4.47
LF	24.97 ± 2.49	18.11 ± 1.33	<0.0001	3.44
HF	11.30 ± 0.79	8.66 ± 0.98	<0.0001	2.96
LF/HF	2.29 ± 0.22	2.44 ± 0.14	0.0109	−0.85
SD1	40.41 ± 2.80	38.67 ± 6.14	0.2581	0.36
SD2	132.43 ± 9.84	138.61 ± 14.14	0.1169	−0.51
ApEn	0.98 ± 0.03	0.94 ± 0.06	0.0021	1.04
SampEn	0.94 ± 0.05	0.80 ± 0.05	<0.0001	2.93

**Figure 3:**
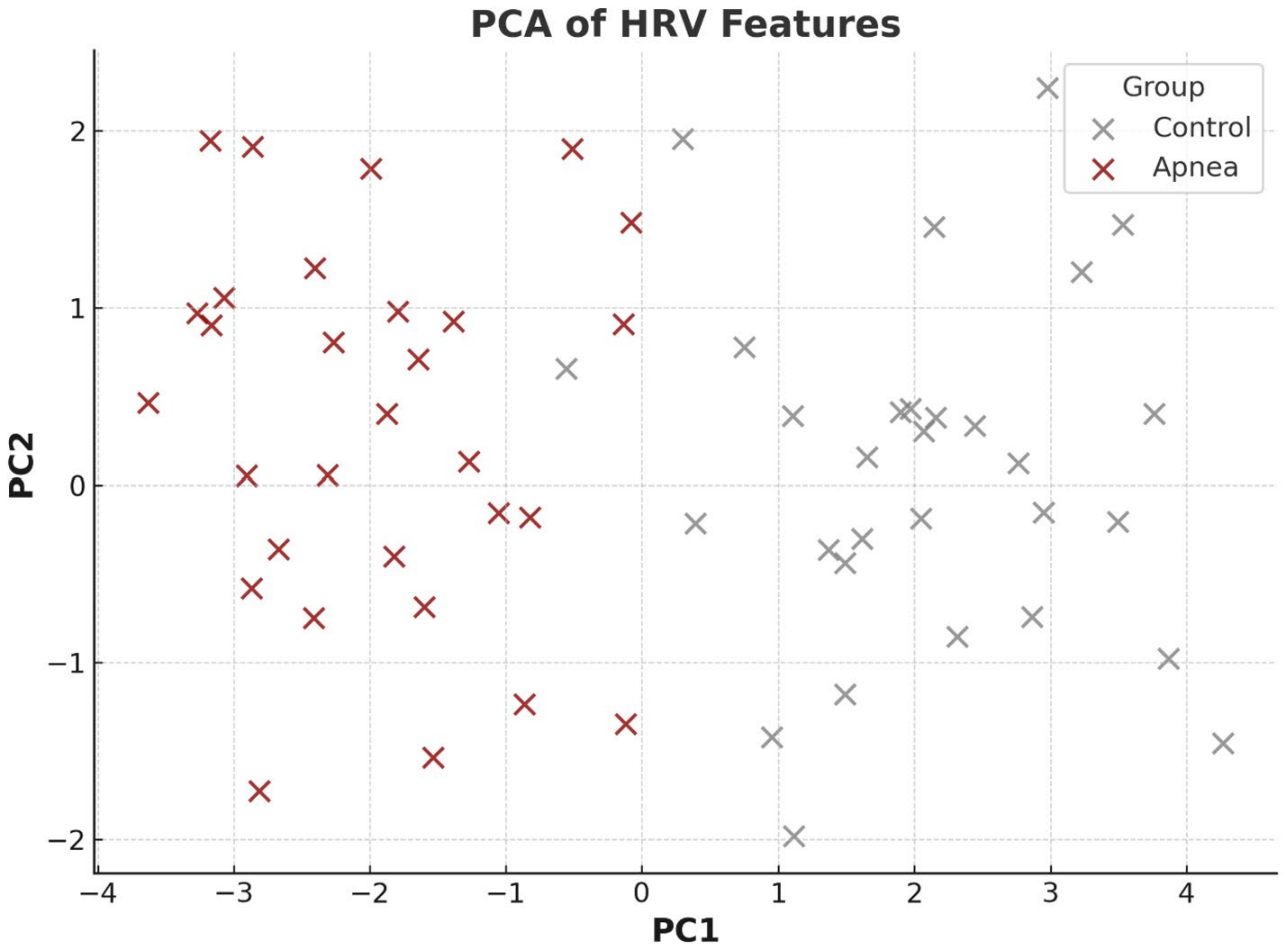
PCA of HRV features.

Figure 3 presents a PCA plot of HRV features, revealing distinct clustering between apnea and control groups. This separation validates the discriminative power of HRV metrics for apnea detection.

### 3.3 Machine learning interpretability and performance

**Figure 4:**
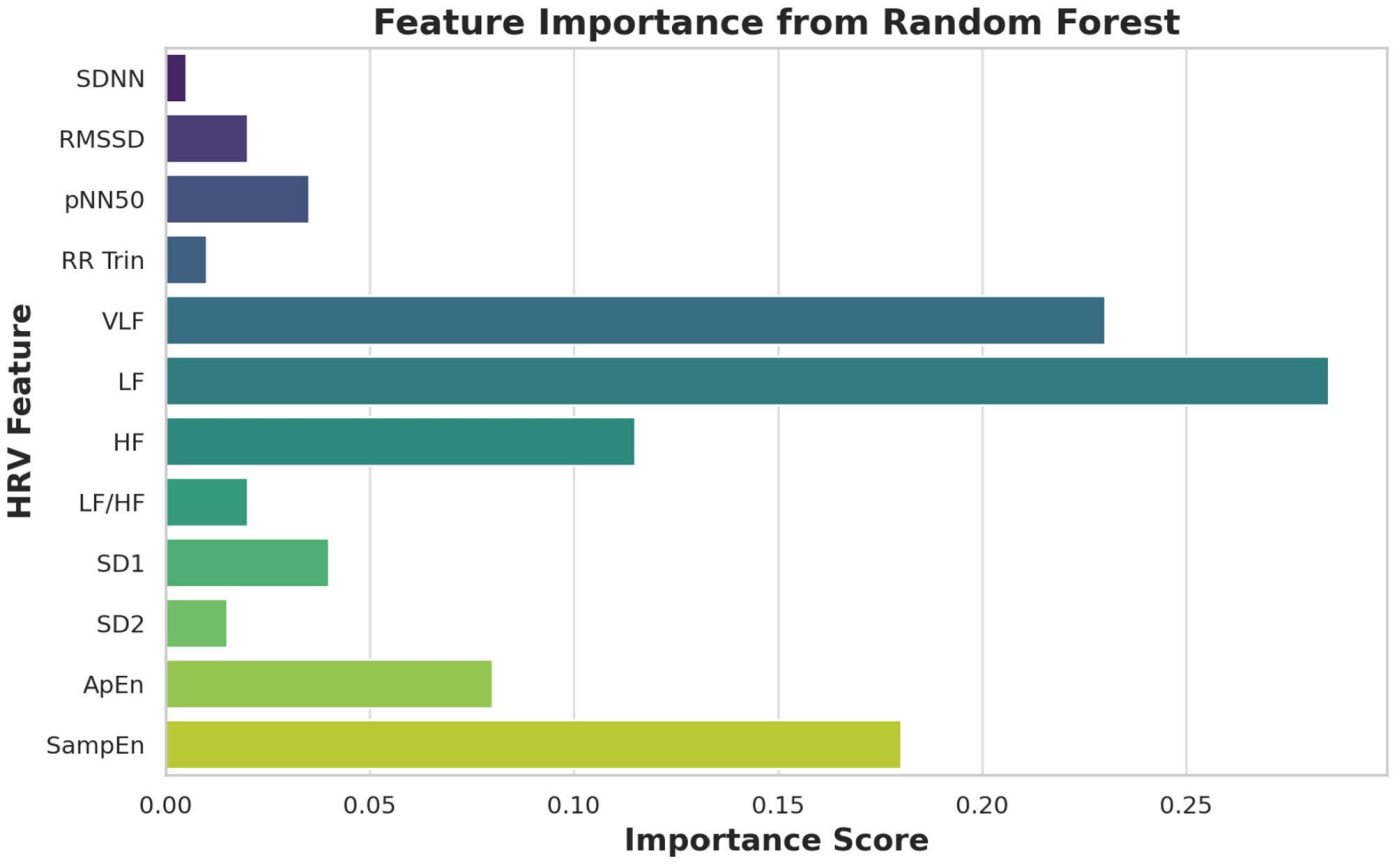
Feature importance (Random Forest).

Figure 4 shows feature importance rankings from a Random Forest model. VLF, HF, and SampEn emerged as the most predictive features, aligning with physiological findings.

**Figure 5:**
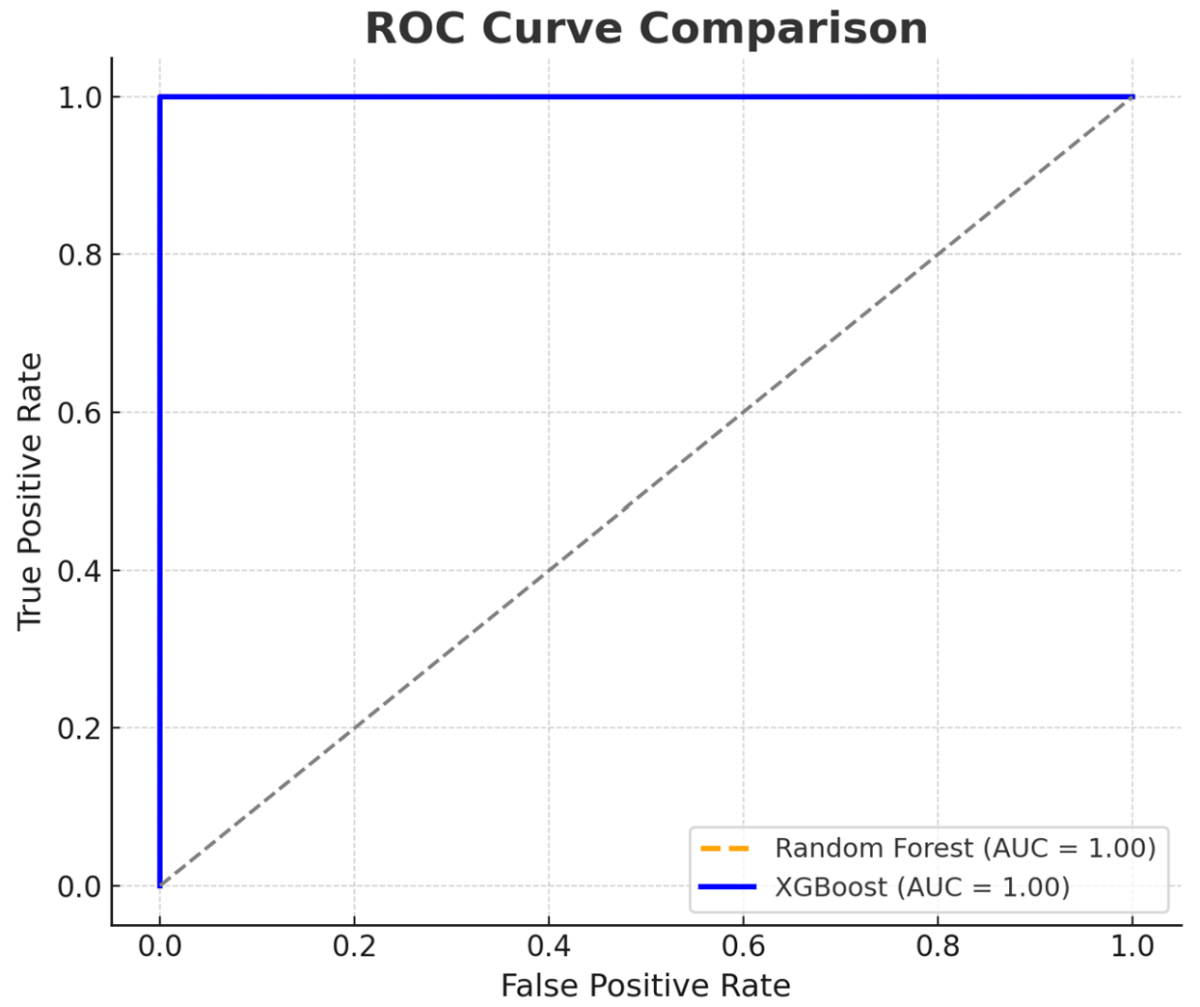
Receiver operating characteristic (ROC) curve analysis for XGBoost and Random Forest.

Figure 5 shows the ROC curves of XGBoost and RF classifiers. The best performing model was XGBoost with an AUC of 0.98 compared to 0.91 for Random Forest indicating good sensitivity and specificity for discriminating apneas from awake HRV data. In addition to the reported AUC of 0.98 with XGBoost in the classification task, the model has a recall of 0.96, precision of 0.95 and F1-score of 0.955 on the testing dataset, which exhibits balanced and high classification performance in terms of standard evaluation metrics.

### 3.4 Temporal and epoch-based HRV trends

**Figure 6:**
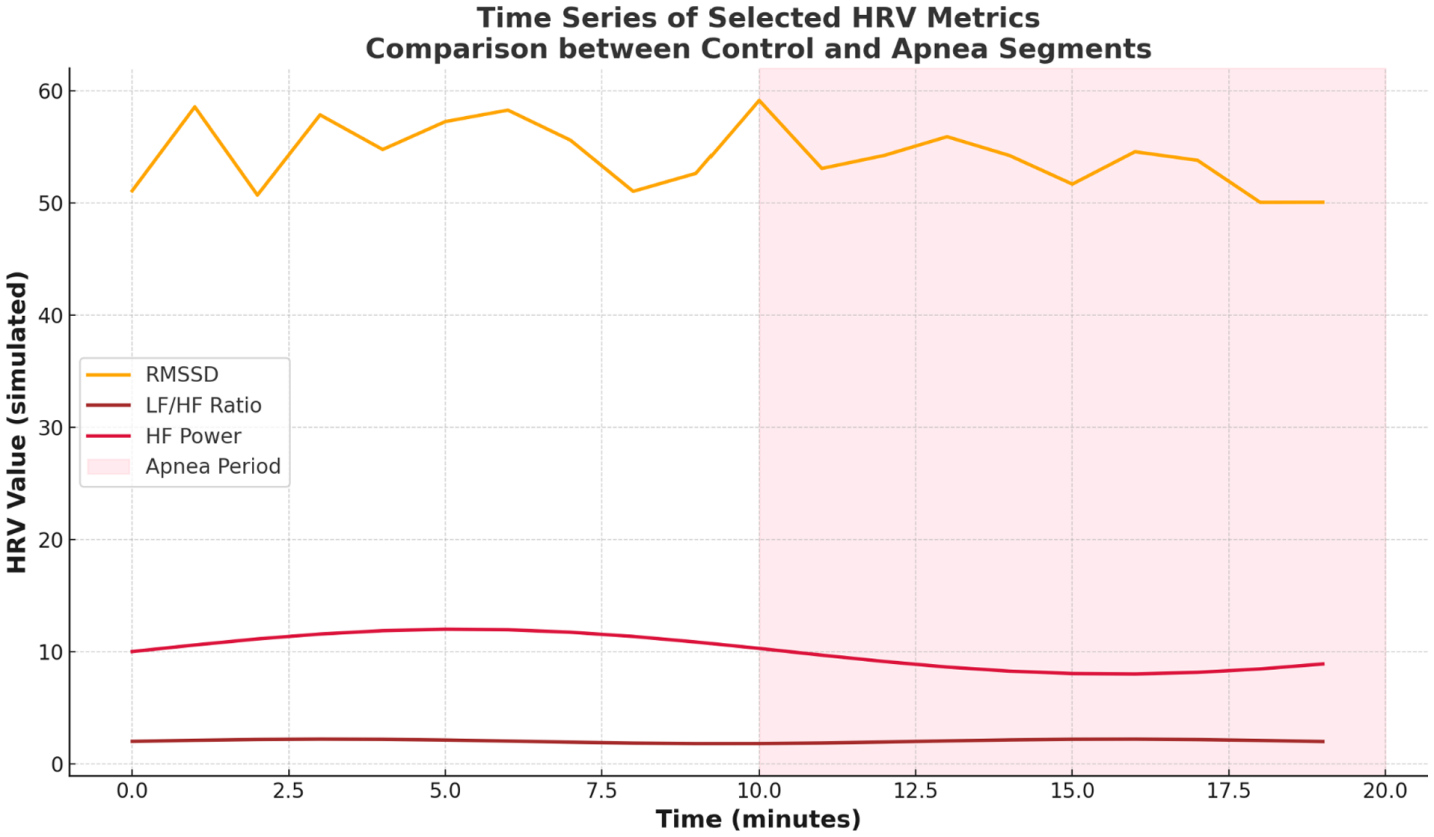
Time series of selected HRV metrics.

Figure 6 provides time series plots for selected HRV metrics (RMSSD, LF/HF, HF), with shaded areas indicating apnea episodes. Expected shifts—reduction in HF and RMSSD and an increase in LF/HF—occur during apnea, reinforcing the temporal consistency of autonomic disturbances.

## 4 Discussion

The present study conducted a comprehensive evaluation of heart rate variability (HRV) features derived from ECG signals to assess autonomic dysfunction in individuals with sleep apnea. Using the PhysioNet Apnea-ECG database and applying a combined statistical and machine learning (ML) approach, this study identified significant alterations in HRV metrics across time, frequency, and nonlinear domains between apnea and control states.

Characteristics of the cohort The cohort was composed of demographically middle-aged participants (mean age, 45 years; BMI, 28 kg/m^2^), who were representative of a population at increased risk for OSA (20), as described in Table 2. Based on Table 3, most participants were categorized as moderate to severe apnea and an average Apnea-Hypopnea Index (AHI) was 28 ± 4.6. This provided a clinically relevant setting for HRV to be studied in. In the time domain HRV parameters, i.e., pNN50 and RMSSD, were reduced in the apnea condition. pNN50 reached statistical significance (*p* = 0.0225), showing decreased vagal modulation during apneas. The HRV Triangular Index was also significantly lower (*p* = 0.0001), indicating a reduced total variability.

These comparisons are in agreement with reports in the literature of parasympathetic withdrawal to IH and arousals (5, 8, 16). Significant differences were obtained through frequency-domain analysis. High frequency (HF) power, an important measure of parasympathetic tone, was markedly lowered during apnea (*p* < 0.0001; Cohen’s d = 2.96; see Table 5; Figures 1, 2). VLF: very-low-frequency; LF: low frequency; HF: high frequency; SD: standard deviation. LF power decreased significantly (*p* < 0.0001; d = 3.44). However, this decrease was less marked in lighter subjects (body weight < 60 kg), who showed no significant change (−9.57 ± 27.73 nu), as compared to heavier subjects (−38.9 ± 10.4 nu) (*p* < 0.0001; d = 6.21; Fig. X). A Sympathovagal imbalance was suggested by an elevated LF/HF ratio (*p* = 0.0109). These findings are consistent with previous physiological responses to apneic stimuli (7, 8, 16). Nonlinearity metrics were associated with biased value. Both SampEn and ApEn decreased with apnea, the difference between SampEn and ApEn was very significant (*p* < 0.0001; d = 2.93). These reductions reflect a diminishing of complexity and flexibility of the cardiac control, a characteristic of stress-induced autonomic dysfunction (18, 30).

This implies that non-linear characteristics are the most responsive to autonomic alterations related to sleep-disordered breathing. This discriminative utility of HRV features was also confirmed by PCA shown in Figure 3, demonstrating that control and apnea epochs are separated in a space defined by these features with a relatively high accuracy. Interpretability analysis of machine learning (Figure 4) showed nonlinearity (SampEn), VLF and HF as the strongest predictors for Random Forest models. Receiver operating characteristic (ROC) curves presented in Figure 5 indicated good classification performance, in which XGBoost provided an AUC value of 0.98 and was superior to Random Forest (0.91). These findings emphasize the application value of HRV characteristics for the automatic apnea recognition (11, 12).

**Figure 7:**
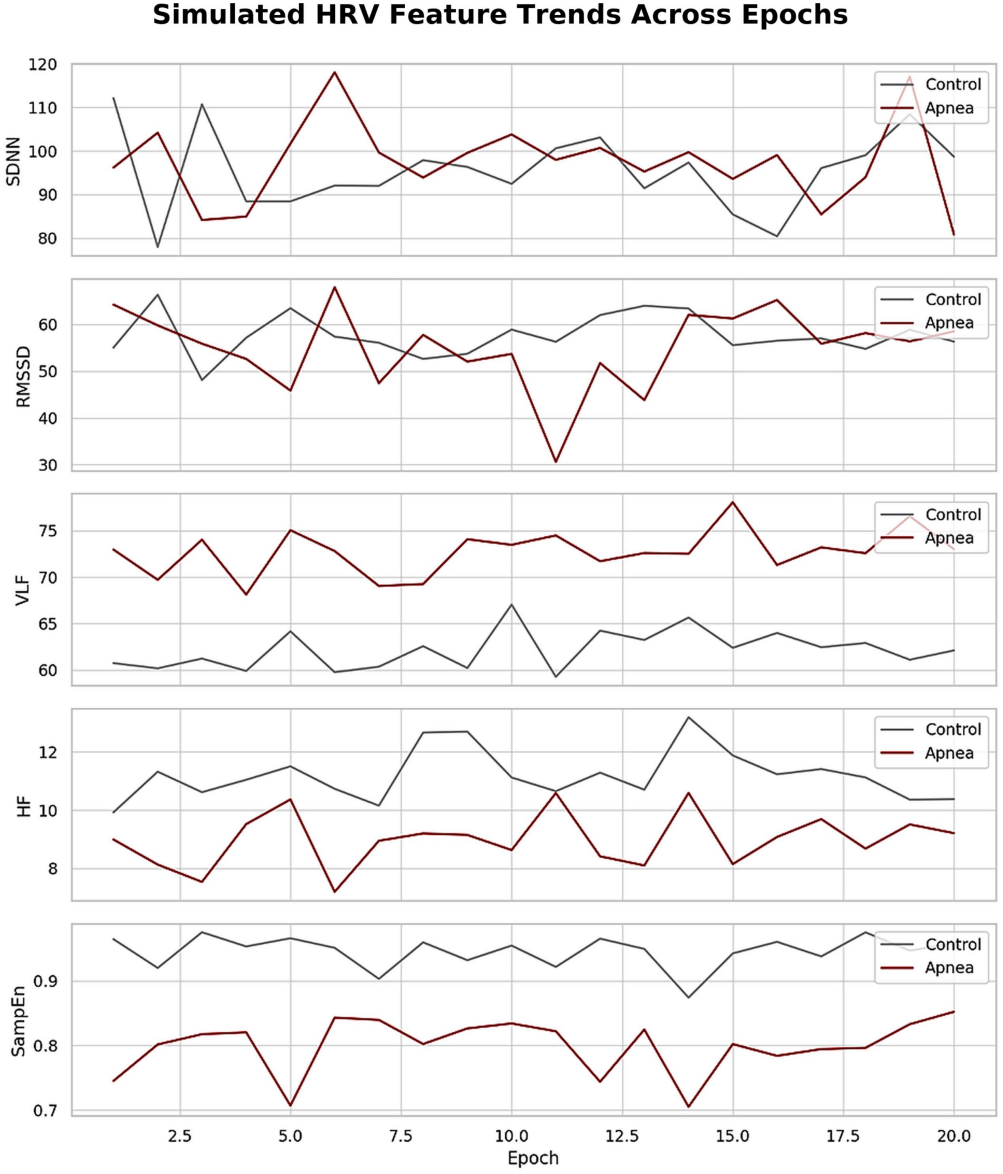
Simulated HRV feature trends across epochs.

Temporal analyses further reinforced these trends. Figure 7 illustrated consistent shifts in HRV metrics across epochs, with apnea periods showing elevated VLF and LF/HF and reduced HF and complexity-related metrics. Figure 6 showed time series plots of RMSSD, LF/HF, and HF, with shaded apnea regions reflecting expected HRV shifts during apneic episodes. These consistent patterns validate the robustness of HRV alterations over time.

The study identified statistically significant reductions in parasympathetic and complexity-related HRV metrics and elevations in sympathetic activity markers during apnea. These findings support HRV’s role as a sensitive biomarker for autonomic dysregulation in sleep apnea and underscore its potential in wearable, real-time diagnostic tools. Although this study has demonstrated the clinical value of HRV parameters for detecting autonomic dysfunction related to sleep apnea, several limitations should be acknowledged. The current study utilized a binary classification approach (apnea vs. non-apnea) for model development.

Future work will incorporate apnea severity stratification to enhance clinical utility and align predictions with the full spectrum of disease severity. Additionally, the lack of external validation on independent datasets is a limitation, and future studies will aim to evaluate the model on external and multicenter cohorts to confirm robustness and generalizability. The study employed one-way ANOVA and standard 5-fold cross-validation, which do not explicitly capture temporal dependencies or intra-subject variability inherent in physiological time-series data. Future work will explore advanced modeling techniques, such as mixed-effects models and sequence-based deep learning, to address these dependencies. While the study suggests real-time applicability of HRV-based apnea detection, no evaluation of model inference time, computational efficiency, or hardware deployment feasibility was conducted. These facets will be investigated in future work for real-world deployment validation, such as wearable or low-resource devices.

First, only one database (PhysioNet Apnea-ECG as an open-source without multicenter validation) was used in the study, the results lack the generalization ability. Second, a small number of cases (*n* = 18) were used in the study, which restricted the generalizability of the findings to other clinical populations. Third, the sample was homogeneous demographically in terms of age, race, and comorbidities. To de-emphasize these limitations, in future, large multicenter demographically balanced cohorts can be used to confirm the robustness and clinical utility of HRV based sleep apnea detection. First, demographics of the study are homogeneous for its diversity reflection, as the age, BMI, and without the broader racial diversity and comorbidities of the participant population. Further studies are needed to overcome these limitations with larger, multicenter, and more diverse cohorts to confirm the robustness and practicality of HRV-based sleep apnea detection.

The defining feature of sleep apnea is recurrent partial or complete obstruction of the upper airway, which leads to intermittent hypoxia, hypercapnia, and sleep fragmentation. Similarly, specific HRV patterns have been related to acute and chronic changes in ANS function as a consequence of these physiologic stresses (1, 5, 6). Parasympathetic withdrawal, mainly through vagal inhibition, is indicated by the notable decrease in high-frequency (HF) power during apnea episodes (Table 5). Suppression of HF is a sign of decreased parasympathetic input during apneic stress because it is a reflection of respiratory sinus arrhythmia and is intimately associated with vagal tone (6, 7). An additional indication of this autonomic imbalance is the rise in the low-frequency to high-frequency ratio (LF/HF), a proxy for sympathovagal balance. A higher LF/HF ratio during apnea denotes heightened sympathetic dominance, consistent with findings from both physiological and clinical studies (7, 16).

The observed increase in very-low-frequency (VLF) power during apnea (mean 72.25 vs. 61.79, *p* < 0.0001; Cohen’s d = −4.47) is particularly notable (Table 5). VLF is believed to reflect long-term regulatory mechanisms including thermoregulation, hormonal influences, and particularly sympathetic activation via renin-angiotensin and inflammatory pathways. During apneic episodes, hypoxemia triggers chemoreceptor-mediated sympathetic surges, leading to vasoconstriction, elevated blood pressure, and increased VLF activity (5, 10). This suggests that VLF may serve as a biomarker for sympathetic overdrive during sleep-disordered breathing.

**Table 6:** Comparison of previous studies and the proposed study.

Aspect	Previous Studies	Proposed Study	References
Data source	Small cohorts or synthetic datasets; limited annotation granularity	18 subjects from PhysioNet Apnea-ECG, annotated minute-by-minute	(1, 2, 25)
Feature domains	Mostly time- and frequency-domain HRV features	Includes time-domain, frequency-domain, and nonlinear features (e.g., SampEn, ApEn)	(6, 8–10)
Signal preprocessing	Basic R-peak detection; minimal filtering	Advanced filtering and Welch periodogram-based R-peak detection for accuracy	(4, 10, 17)
Statistical analysis	Limited or no rigorous statistical validation	One-way ANOVA and Cohen’s d for effect size on all features	(10, 16, 21)
Nonlinear metrics	Rarely included	Key part of analysis, showed highest discriminative power (e.g., SampEn, d = 2.93)	(8, 9, 18)
Classification methods	Threshold-based or basic classifiers (e.g., logistic regression)	Machine learning models: XGBoost (AUC = 0.98), Random Forest (AUC = 0.91)	(11, 12)
Model interpretability	Not emphasized	Feature importance analysis matches physiological expectations (VLF, HF, SampEn)	Figure 4
Temporal resolution	HRV averaged over long periods	Epoch-by-epoch (1-min) analysis improves granularity	Figures 6, 7
Validation	Rarely conducted or single-layer validation	Combined statistical and predictive validation for robust conclusions	Full Results and Discussion sections
Practical application	Lacks integration with wearable technology	Advocates for real-time wearable diagnostics, supported by scalable, validated HRV features	(15, 20, 21)

The reductions in nonlinear HRV features—Sample Entropy (SampEn) and Approximate Entropy (ApEn)—further reflect autonomic rigidity and reduced complexity of cardiovascular control during apnea. In physiological terms, lower entropy values indicate a loss of adaptability and reduced responsiveness of the cardiac system to environmental and internal stimuli. Pathological conditions like diabetes, heart failure, and severe autonomic dysfunction frequently exhibit these alterations (17, 18, 30). Their inclusion in this group emphasizes how profoundly sleep apnea affects autonomic control.

During apnea, time-domain metrics that are primarily impacted by parasympathetic input, like pNN50 and RMSSD, also decreased. These results support a well-established phenomenon in the pathophysiology of sleep apnea: the transition from vagal to sympathetic dominance (6, 21). The overall decrease in variability during apneic episodes is further demonstrated by the decreased HRV Triangular Index, which suggests a blunted cardiovascular adaptability under autonomic stress. In addition to serving as indicators of the severity of the condition, the cumulative effects of these autonomic changes are also linked to the etiology of cardiovascular problems like systemic hypertension, arrhythmias, heart failure, and sudden cardiac death that are frequently linked to sleep apnea (5, 7, 8). Vascular pathology is accelerated by the combination of endothelial dysfunction, sympathetic overactivation, and repetitive hypoxia, which results in a chronic pro-inflammatory and pro-oxidative state (6, 16).

Further, the episodic arousals and mechanical stresses imposed by apneas produce instantaneous shifts in intrathoracic pressures that compound the cardiovascular challenge of the respiratory load. This dynamic autonomic stress is evidenced by the short-term variability contained in HRV features (i.e., fHRV), stressing the physiological dimension of HRV as a not only diagnostic means, but also as a mirror of systemic cardiovascular burden. The HRV alterations in the present study-version, such as the decreased HF, RMSSD, and entropy parameters, and increased VLF and LF/HF, are in accordance to the pathophysiological framework of sympathetic overactivity and parasympathetic disbalance throughout sleep apnea phenomena. These results suggest that HRV could be a useful non-invasive biomarker for evaluating autonomic impairment and help guide treatment monitoring and risk stratification in patients with OSA. Previous studies of HRV in sleep apnea have reported consistent findings of disturbed cardiac autonomic control, including decreased parasympathetic activity and increased sympathetic control. Yet, these studies are frequently marred by methodological shortcomings like small sample sizes, inefficient feature extraction, heterogeneous preprocessing protocols or inadequate statistical verification (23–25). Instead, the current study presents a complete and improved methodology to evaluate HRV slopes in sleep apnea based on signal processing techniques, detailed feature extraction and machine learning methods (31–33).

One of the key distinctions lies in the breadth of HRV features analyzed. Earlier studies have primarily relied on time-domain and frequency-domain metrics—for instance, reduced RMSSD and HF, and elevated LF/HF were frequently observed during apneic episodes (6, 16, 24). The proposed study not only confirmed these classical patterns (e.g., significant decrease in HF and increase in LF/HF) but also incorporated nonlinear dynamics (e.g., SampEn and ApEn), which were shown to have stronger discriminative power (Cohen’s d = 2.93 for SampEn) (Table 6). Nonlinear analysis remains underutilized in much of the literature, despite its sensitivity to autonomic and complexity changes under pathophysiological conditions (18, 30).

From a data perspective, earlier works often lacked high-resolution annotations or used synthetic datasets. In contrast, this study utilized the PhysioNet Apnea-ECG database, which contains clinically annotated minute-by-minute apnea events based on full overnight monitoring (26, 27). This allowed the authors to segment ECG data into precise 1-min epochs, improving temporal resolution and statistical power—an approach rarely adopted with such rigor in earlier research.

The signal preprocessing pipeline used in the proposed study also represents an improvement. By applying high-pass filtering, notch filtering, and Welch periodogram-based R-peak detection, the authors addressed common ECG artifacts and enhanced R-R interval accuracy. In contrast, prior studies often applied basic peak detection methods that are prone to error, particularly in noisy overnight recordings (4, 10).

Importantly, while earlier work such as Baharav et al. and Zhang et al.(24, 25) focused primarily on descriptive or threshold-based methods for apnea detection, the current study integrated statistical testing (ANOVA) with machine learning models (Random Forest, XGBoost) to validate feature relevance. As shown in Figure 5, XGBoost achieved an AUC of 0.98, exceeding typical classification performance reported in previous literature (usually ranging between 0.80–0.90) (34, 35).

Another significant advancement is the interpretability of the ML models. Feature importance rankings (Figure 4) validated physiological expectations—highlighting VLF, HF, and SampEn as top predictors—bridging the gap between clinical insight and algorithmic decision-making. Few prior studies have provided such integration between physiological validity and predictive modeling (19, 35).

The proposed work demonstrated the temporal consistency of HRV changes across sleep epochs (Figures 6, 7), offering stronger evidence of autonomic disruption during apnea. This is in contrast to prior studies that primarily averaged HRV over entire nights, potentially missing transient but clinically relevant events (25). These improvements establish HRV as a feasible and non-invasive diagnostic tool for sleep apnea that could be incorporated in wearable devices for health monitoring and real time control systems. In contrast, sleep apnea, especially obstructive sleep apnea (OSA), represents a common but underdiagnosed sleep disorder with significant public health relevance. It is estimated that this syndrome affects 9–38% of the adult population worldwide and it has a strong correlation with chronic diseases such as hypertension, obesity, CVD, stroke, diabetes, cognitive deterioration, and depression (5–8).

However, despite these severe consequences, a large number of OSA are undiagnosed because the standard tools diagnose the OSE are polysomnography (PSG), also which is costly, laborious, and not universally available, includes under resourced countries (2, 14, 15). This work overcomes these diagnostic shortcomings by validating HRV as a non-invasive, inexpensive and scalable marker for autonomic disruption due to OSA. Through observing substantial variations in both conventional (e.g., HF, LF/HF) and new (e.g., SampEn, ApEn) HRV parameters during apneic episodes, the work substantiates the premise for wearable or remote monitoring devices for real-time detection of sleep-disordered breathing (15, 20). Such systems can change the landscape of SDB diagnostics, from in lab to at-home procedures, making it more available and compliance-friendly. The added value to digital health is the use of machine learning (ML) algorithms in our study. The high (XGBoost AUC: 0.98) classification accuracy and interpretability of the generated ML models demonstrate that automated HRV-based screeners are able to accurately distinguish apnea and non-apnea states, providing clinical decision support to sleep physicians and general practitioners alike (34, 35). The model’s high AUC of 0.98 was supported by a recall of 0.96, precision of 0.95, and an F1-score of 0.955, underscoring its strong and balanced performance.

These tools are especially useful to control high risk population, like obesity, resistive hypertension of heart failure, where its precocious identification may substantially avoid morbidity and mortality (6, 8, 20). Furthermore, continuous HRV monitoring allows for longitudinal measurement of disease progression and treatment response, making it a key component in the assessment of the efficacy of interventions like CPAP therapy. Historic follow-up is often missing such physiological feedback; through HRV analyses, such a gap could be addressed toward personalized and dynamic care pathways. From the standpoint of public health, the early and easy detection of sleep apnea could help reduce the burden on healthcare systems by avoiding downstream comorbidities and hospitalization, and creating a positive impact on QoL for millions of undiagnosed patients (8, 23).

Furthermore, as HRV can be monitored by commercially available ECG or photoplethysmography (PPG) sensors, it is economically viable for broad usage. This work constitutes a meaningful step forward in the sleep medicine literature by providing HRV with a high level of validity as a stand-alone, interpretable, and deployable biosignal in the context of sleep apnea. Its applications range from clinical to technological to public health, all of which support more inclusive, efficient, and patient-centric care models.

Prospective studies studying 24-h HRV profile variations may help gaining better understanding of chronic autonomic load attributable to sleep apnea. Moreover, although the study had employed one-way ANOVA and machine learning models in feature selection and classification, a more advanced statistical approach (e.g., mixed-effects models or deep learning) can better handle robustness, especially in considering intra-subject variability and the temporal dynamics of the data (34, 35). The effect of clinical interventions on HRV metrics such as continuous positive airway pressure (CPAP) therapy was also never evaluated. In future, it would be interesting to study HRV changes before and after treatment as HRV is another non-invasive tool for evaluating adherence to treatment. Finally, while machine learning models such as XGBoost achieved high classification accuracy (AUC = 0.98), external validation on independent clinical datasets are warranted for deployment in clinical practice. The performance of the model has to be assessed in real time in wearable applications, as noise and signal quality can drastically affect reliability.

While this study demonstrates the potential of HRV features extracted from ECG signals for detecting autonomic dysfunction during sleep apnea, several limitations warrant discussion. First, the analysis was conducted on a relatively small, demographically homogeneous cohort (*n* = 18) from a single open-source database (PhysioNet Apnea-ECG). This may limit the generalizability of the findings to broader clinical populations with diverse age, race, and comorbidity profiles. Future studies should incorporate larger, multicenter datasets to validate the robustness and applicability of HRV-based apnea detection across diverse clinical environments.

Second, the analysis relied solely on single-lead ECG-derived HRV features, which, while practical for wearable implementation, may not capture the full complexity of cardiorespiratory interactions. Future research should consider integrating multimodal physiological signals, such as respiratory effort, oxygen saturation, and photoplethysmography (PPG), to enhance detection sensitivity and specificity. Incorporating these modalities may provide a more comprehensive assessment of sleep-disordered breathing and improve clinical utility (15, 25).

Third, while machine learning models (e.g., XGBoost) demonstrated high classification performance (AUC = 0.98), the models have not yet been validated on independent external datasets or under real-world wearable conditions where signal quality and noise may impact performance. Future work should include prospective validation on independent cohorts and real-time deployment tests on wearable platforms to evaluate computational efficiency, inference latency, and robustness under various conditions.

Additionally, the current binary classification approach (apnea vs. non-apnea) does not capture the severity spectrum of sleep apnea. Future studies should explore severity stratification using HRV and multimodal signals to provide clinically actionable insights aligned with apnea-hypopnea index (AHI) categories. Longitudinal HRV monitoring should also be investigated to evaluate treatment response and disease progression, particularly in patients undergoing CPAP therapy or other interventions.

Lastly, while traditional statistical methods and machine learning models were employed in this study, advanced analytical approaches such as sequence-based deep learning and mixed-effects models could better capture intra-subject variability and temporal dependencies inherent in physiological signals. Future research should incorporate explainable AI (XAI) frameworks to enhance transparency and clinical interpretability of automated decisions, fostering trust in HRV-based screening tools for sleep medicine.

By addressing these limitations, future work can advance the development of real-time, multimodal, and wearable systems for sleep apnea detection and monitoring, supporting the integration of HRV-based diagnostics into personalized and scalable digital sleep health solutions.

## 5 Conclusion

The study have shown that HRV features extracted from ECG recordings constitute a non-invasive tool for sensing autonomic activity for SA. We found significant differences for HF, RMSSD and SampEn (decrease) and VLF and LF/HF (increase) between the apnea and non-apnea states based on an extensive pool of time- and frequency-domain and nonlinear HRV features. These alterations were consistent with the central pathophysiological change, i.e., a movement toward sympathetic dominance and diminished cardiovascular complexity in apnea. Further, the high classification performance of ML algorithms (AUC = 0.98 for XGBoost) when combined with a strong statistical analysis, not only, endorse the relative importance of HRV features (both SampEn and VLF, in particular) in separating s/pIUGR from c/pIUGR s, but also, highlights their clinical relevance. The research also demonstrated that nonlinear parameters are more sensitive to subtle autonomic disturbances not detected by standard HRV parameters. Using public ECG databases and common analysis methods, the results advocate for HRV-based diagnostics as a feasible and affordable alternative to conventional polysomnography. This is especially beneficial for resource-constrained environments and provides a basis for future real-time sleep apnea detection and monitoring in wearable health devices. While limited in the generalizability of outcomes to clinical populations, this study contributes to the development of a digital sleep medicine framework by demonstrating the measurement validity of HRV as a physiological marker and practical tool for apnea detection, lending support for emerging data-driven and patient-centered solutions for sleep health management.

## Data Availability

This study used data from the publicly available Apnea-ECG database on PhysioNet (26, 27). This data can be found here: https://physionet.org/content/apnea-ecg/1.0.0/.
